# Oral Gepotidacin for the Treatment of Uncomplicated Urogenital Gonorrhea: Nucleic Acid Amplification Testing Outcomes in a Randomized, Multicenter Phase 3 Trial (EAGLE-1)

**DOI:** 10.1093/ofid/ofag438

**Published:** 2026-07-17

**Authors:** William G Flight, Janet Wilson, David A Lewis, Jonathan D C Ross, Sally Gatsi, Charles Jakielaszek, Dan T Lythgoe, Salim Janmohamed, Judith Absalon, Matthew Helgeson, Caroline Perry

**Affiliations:** GSK, London, UK; The Leeds Teaching Hospitals NHS Trust, Leeds, UK; Sydney Medical School—Westmead, University of Sydney, Sydney, New South Wales, Australia; Western Sydney Local Health District, Western Sydney Sexual Health Centre, Sydney, New South Wales, Australia; University Hospitals Birmingham NHS Foundation Trust, Birmingham, UK; Organon LLC, Jersey City, New Jersey, USA; GSK, Collegeville, Pennsylvania, USA; GSK, Stevenage, UK; GSK, London, UK; GSK, Collegeville, Pennsylvania, USA; GSK, Collegeville, Pennsylvania, USA; GSK, Collegeville, Pennsylvania, USA

**Keywords:** EAGLE-1, gepotidacin, gonorrhea, NAAT, *Neisseria gonorrhoeae*

## Abstract

**Background:**

The phase 3 EAGLE-1 trial demonstrated noninferiority of oral gepotidacin to ceftriaxone plus azithromycin for treating uncomplicated urogenital gonorrhea. To reflect real-world practice, we conducted an exploratory analysis of efficacy assessed by nucleic acid amplification tests (NAAT).

**Methods:**

EAGLE-1 (NCT04010539) compared the efficacy and safety of oral gepotidacin (2 × 3000 mg) with intramuscular ceftriaxone (1 × 500 mg) plus oral azithromycin (1 × 1000 mg) in participants (≥12 years) with uncomplicated urogenital gonorrhea. Urogenital specimens for NAAT were collected at baseline and test-of-cure (days 4–8). The microbiological intent-to-treat (micro-ITT) population comprised all participants who received ≥1 dose of study treatment, with culture-confirmed ceftriaxone-susceptible urogenital *Neisseria gonorrhoeae* at baseline. The microbiologically evaluable NAAT test-of-cure population comprised micro-ITT participants with valid baseline NAAT-positive results, who followed important trial protocol components and valid test-of-cure NAAT results. NAAT–confirmed treatment success was defined as nucleic acid clearance at the test-of-cure.

**Results:**

High concordance between NAAT and culture results was observed (98.4%). In the micro-ITT population, NAAT-confirmed success rates at the test-of-cure were 82.5% (151/183) for gepotidacin and 75.0% (132/176) for ceftriaxone plus azithromycin (adjusted difference 8.2%, 95% confidence interval [CI], −0.1%, 16.5%); numerically lower than the corresponding culture-confirmed success rates of 92.6% (187/202) and 91.2% (186/204). In the microbiologically evaluable NAAT test-of-cure population, NAAT-confirmed success rates at the test-of-cure were 88.3% (151/171) for gepotidacin and 86.3% (132/153) for ceftriaxone plus azithromycin (adjusted difference 3.8%, 95% CI, −3.6%, 11.2%).

**Conclusions:**

The NAAT-confirmed success rates were high and similar between treatments, supporting the primary analysis results based on culture.

## Teaser

Urogenital gonorrhea treatment success rates by NAAT were high and similar between gepotidacin and ceftriaxone plus azithromycin, supporting primary analysis results based on microbiological culture. Therefore, NAAT may be a promising complement to culture in future clinical trials.

Gonorrhea is one of the most common sexually transmitted infections worldwide, with ∼85 million new cases annually in adolescents and adults [1–3]. The infection, which may present asymptomatically, is caused by the bacterium *Neisseria gonorrhoeae*, which primarily causes urogenital disease but may occur in the rectum and pharynx [1–3]. Untreated gonorrhea can cause serious and permanent health problems [1, 2, 4]. Treatment options are limited due to rising antimicrobial resistance among *N. gonorrhoeae* strains, posing the risk of complications and serious implications for public health worldwide [5]. Currently, intramuscular ceftriaxone with or without azithromycin is the recommended empirical outpatient treatment by the World Health Organization (WHO), the International Union against Sexually Transmitted Infections, and the Centers for Disease Control and Prevention [6–8]. New antimicrobial agents to manage gonorrhea, particularly antibiotic-resistant strains, are urgently needed. Additionally, oral therapies offer advantages over injectable treatments, including improved accessibility and ease of use in diverse clinical settings [9].

Gepotidacin is an oral, first-in-class, bactericidal, triazaacenaphthylene antibacterial that inhibits bacterial DNA replication by a distinct binding site on GyrA and ParC and a unique mechanism of action [10, 11]. Gepotidacin was approved in 2025 in the United States and the United Kingdom for the treatment of uncomplicated urinary tract infections in female patients aged ≥12 years and weighing ≥40 kg, based on the results of 2 large pivotal phase 3 trials [12–14]. Results of the recent multinational EAGLE-1 phase 3, open-label (sponsor-blinded), active-comparator, noninferiority trial demonstrated that gepotidacin was noninferior to first-line ceftriaxone plus azithromycin for the treatment of urogenital *N. gonorrhoeae* [15]. Bacterial persistence of urogenital gonorrhea was not seen with either treatment, and no new safety concerns with gepotidacin were identified. Gepotidacin was approved in December 2025 by the US Food and Drug Administration (FDA) for the treatment of uncomplicated urogenital gonorrhea in adult and pediatric patients aged ≥12 years and weighing ≥45 kg [12].

Microbiological testing is integral for the accurate and timely diagnosis of gonorrhea and is essential for effective treatment and transmission prevention [16]. Techniques include microscopy of Gram-stained samples, microbiologic culture, and, commonly today, nucleic acid amplification tests (NAATs) to detect pathogen-specific DNA or RNA. Regulatory agencies, including the FDA, mandate microbiologic culture confirmation for the clinical evaluation of new treatments for gonorrhea [17], as was required in the EAGLE-1 trial of gepotidacin. Culture confirms viable *N. gonorrhoeae* presence and offers extensive phenotypic antimicrobial susceptibility testing [4–7], critical for resistance surveillance and assessing therapeutic efficacy beyond microbiological eradication alone [4, 5, 15]. Nucleic acid amplification tests provide rapid detection and, for certain antibiotics, predict susceptibility phenotype [18]. Nucleic acid amplification tests offer greater practicality and higher sensitivity (>95%) in both symptomatic and asymptomatic infections compared with culture, the sensitivity of which varies greatly (up to 95% for urogenital, symptomatic infections under optimal conditions; ∼50%–85% for extragenital or asymptomatic infections) [5, 6, 19, 20]. However, NAATs have a variable specificity which is generally lower than culture and, therefore, have a higher risk of incorrect interpretation of results. For example, a positive result at a test-of-cure may suggest bacterial persistence when, in fact, it is due to the detection of residual nucleic acid postbacterial clearance [4].

A prior nonregistrational clinical trial, evaluating the antibiotic gentamicin for the treatment of gonorrhea, used NAATs to assess treatment efficacy, alongside culture to assess antimicrobial susceptibility and establish breakpoints [21]. This approach reflects the increasing use of NAATs in standard clinical practice guided by recommendations from US [4], UK [5], and European [7] organizations, and the WHO [6], which endorse microbiologic culture and/or NAATs for the diagnosis of gonorrhea.

To enhance the relevance of the primary efficacy results of the EAGLE-1 trial in the context of real-world clinical practice in many countries, we conducted a prespecified exploratory analysis of gepotidacin and ceftriaxone plus azithromycin efficacy assessed by NAAT.

## METHODS

### Study Design

A full description of the study methodology is available in the primary publication [15]. Briefly, EAGLE-1 (NCT04010539) was a phase 3, open-label (sponsor-blinded), multicenter, active comparator-controlled, noninferiority study conducted between October 2019 and October 2023. The study compared the efficacy and safety of oral gepotidacin (two 3000 mg doses administered 10–12 hours apart) with intramuscular ceftriaxone (500 mg) plus oral azithromycin (1 g) as single doses in participants with uncomplicated urogenital gonorrhea.

The study was conducted in accordance with the ethical principles of the Declaration of Helsinki and the International Council for Harmonisation Guidelines for Good Clinical Practice. The study protocol was approved by the relevant research ethics committees and institutional review boards, as appropriate for participating study centers.

### Participants

Eligible participants were aged ≥12 years, weighed >45 kg, and had clinical suspicion of a urogenital gonococcal infection, with any of the following: symptomatic discharge (urethral in men and endocervical or vaginal in women), recent genital discharge with microscopically visualized intracellular diplococci (≤5 days before screening), or a positive, *N. gonorrhoeae* result by culture (≤5 days before screening) or NAAT (≤7 days before screening), as long as the participant has not received any treatment for this infection. Participants were also required to have retained their original urogenital anatomy from birth. Full details of participant eligibility have previously been described [15]. All participants provided written informed consent prior to study entry.

### NAAT Assay

Pretreatment urogenital specimens were obtained from all participants at baseline for NAAT assay to detect the presence of urogenital *N. gonorrhoeae* nucleic acid, conducted at a central laboratory (PPD GCL, Highland Heights, KY, United States). In addition, pretreatment baseline rectal and pharyngeal specimens for the NAAT assay for the detection of *N. gonorrhoeae* were collected optionally from participants who consented. *Neisseria gonorrhoeae* testing was conducted using cobas CT/NG v2.0 test with the cobas system 4800 (F. Hoffman-La Roche Ltd, Basel, Switzerland).

A test-of-cure visit was performed between days 4 and 8. At the test-of-cure visit, specimens were collected for NAAT assay for detection of *N. gonorrhoeae* from all body sites sampled at baseline. Only participants who had *N. gonorrhoeae* nucleic acid detected from their baseline central laboratory NAAT specimen were evaluated for NAAT outcome and response. At a follow-up visit (days 14–21, following study initiation), a pharyngeal specimen was collected for detection of *N. gonorrhoeae* from participants who had a positive NAAT assay for pharyngeal *N. gonorrhoeae* at both baseline and test-of-cure and who had not received other systemic antimicrobials prior to the follow-up assessment. Further details of sample collection and assessments of culture and NAAT are provided in Supplementary Tables 1 and 2, respectively.

### Efficacy: Microbiological Success

The primary efficacy endpoint in EAGLE-1 was microbiological success, defined as culture-confirmed bacterial eradication of *N. gonorrhoeae* from the urogenital body site at the test-of-cure visit. Secondary efficacy endpoints included culture-confirmed bacterial eradication of *N. gonorrhoeae* at the rectal and pharyngeal sites.

Microbiological success was also measured via NAAT assay for several exploratory endpoints. Microbiological outcome and treatment response were determined by a prespecified and programmed algorithm that compared baseline NAAT results with those obtained at the test-of-cure (and follow-up where applicable) visits. A post hoc analysis was conducted to assess NAAT-determined treatment success by day for the test-of-cure visit performed within the prespecified analysis visit window (days 4–10). Clearance of baseline *N. gonorrhoeae* nucleic acid was assessed in urogenital specimens at the test-of-cure visit. Criteria for defining microbiological outcome and treatment response by NAAT at the test-of-cure are found in Table 1.

**Table 1.** Defining Criteria for Microbiological Outcome and Treatment Response at Test-of-Cure Determined by NAAT

Defining Criteria	Microbiological Outcome	Treatment Response
Clearance of baseline pathogen nucleic acid from a specimen taken at the test-of-cure, without the participant receiving other systemic antimicrobials before this visit	Nucleic acid clearance	Treatment success
Persistence of baseline pathogen nucleic acid from a specimen taken at the test-of-cure, without the participant receiving other systemic antimicrobials before this visit	Nucleic acid persistence	Treatment failure^a^
A determination of the nucleic acid outcome from a specimen taken at the test-of-cure cannot be made (eg, no sample taken, sample lost, visit did not occur) The participant received other systemic antimicrobials before test-of-cure	UTD	Treatment failure^a^

Specimen refers to either a urogenital, pharyngeal, or rectal specimen obtained for NAAT. The microbiological outcomes are determined on a per-pathogen and per-specimen basis.

Abbreviations: NAAT, nucleic acid amplification test; UTD, unable to determine.

^a^Participants who were a “Treatment failure” due to persistence at the test-of-cure assessment, yet did not receive other systemic antimicrobials, were evaluable at subsequent time points.

### Analysis Populations

Details for study analysis populations are reported in the primary publication [15]. Here, we report data for 3 main populations: the intent-to-treat (ITT) population (all participants who were randomly assigned to a study treatment), the microbiological ITT (micro-ITT) population (all randomized participants who received at least one dose of study treatment and had culture-confirmed ceftriaxone-susceptible urogenital *N. gonorrhoeae* at baseline), and the microbiologically evaluable NAAT test-of-cure population. This third population comprised participants who met the definition of the micro-ITT population, who were *N. gonorrhoeae* NAAT positive at baseline, and who followed important components of the study (ie, [1] received all planned doses as randomized, and actual treatment was the same as randomized; [2] had a urogenital NAAT specimen collected at the test-of-cure, with available NAAT results; [3] had not taken any nonstudy systemic antimicrobial prior to test-of-cure [unless taken for the current infection]; and [4] had no major protocol deviations that prevented evaluation of efficacy).

Inclusion criteria and analysis methods for the micro-ITT rectal, micro-ITT pharyngeal, and microbiological evaluable NAAT test-of-cure populations are described in Supplementary Methods.

### Statistical Analysis

Treatment success rates determined by NAAT at the test-of-cure were estimated with accompanying 95% Exact Clopper–Pearson confidence intervals (CIs) for each treatment group. For the urogenital site, the difference in treatment success rates by NAAT between the 2 treatments and corresponding 2-sided 95% CI were estimated using the Miettinen–Nurminen score method [22], adjusting for actual sex and sexual orientation strata (men who have sex with women; men who have sex with men, including bisexual male participants who reported sex with both men and women; or female) [23].

Concordance between central laboratory culture identification and central laboratory *N. gonorrhoeae* NAAT at baseline was assessed by classical diagnostic test statistics (concordance, sensitivity, and positive predictive value) in participants with both culture and NAAT data at baseline for the urogenital site (post hoc analysis). Too few urogenital *N. gonorrhoeae*–negative samples at baseline were available at the central laboratory to assess specificity.

## RESULTS

### Study Disposition and Demographic and Clinical Characteristics

Full details of participant disposition were reported in the EAGLE-1 study primary publication [15]. Of 628 participants enrolled in the study, 406 (64.6%) participants (202 in the gepotidacin group and 204 in the ceftriaxone plus azithromycin group) were included in the micro-ITT population. The microbiologically evaluable NAAT test-of-cure population included 324 (51.6%) participants, comprising 171 and 153 participants in the gepotidacin and ceftriaxone plus azithromycin groups, respectively.

Demographic and clinical characteristics for the micro-ITT population have previously been reported [15] and were generally balanced across both treatment groups. Of the 406 participants in the micro-ITT population, most participants were male (372, 91.6%) and the age of participants ranged from 17 to 64 years, with a mean age of 33.1 years.

### Pathogen Detection Rates

Similar pathogen detection rates at the urogenital site by NAAT were observed at baseline for the 2 treatment groups (Table 2). Baseline concordance between central laboratory NAAT and culture results for urogenital *N. gonorrhoeae* was 98.4%, with a sensitivity of 99.4% and positive predictive value of 98.9% (post hoc analysis, Supplementary Table 3).

**Table 2.** NAAT Assay Results for *Neisseria gonorrhoeae* at Baseline at the Urogenital Site (ITT Population)

Pathogen	Gepotidacin 2 × 3000 mg (N = 314)	Ceftriaxone 500 mg Plus Azithromycin 1 g (N = 314)	Total (N = 628)
n^a^	314	314	628
*N. gonorrhoeae*			
Positive	213 (68)	210 (67)	423 (67)
Negative	63 (20)	71 (23)	134 (21)
Missing^b^	38 (12)	33 (11)	71 (11)

Abbreviations: ITT, intent-to-treat; NAAT, nucleic acid amplification test.

^a^Percentages were calculated from n. Data are presented for NAAT assay results at baseline based on PPD GCL (ie, central laboratory) results. Data are n (%).

^b^Missing category included samples not collected or not received by the PPD GCL or any specimens that were submitted to the PPD GCL but whose results were not available (ie, improper sample submission).

### Urogenital Site: Treatment Efficacy by NAAT at Test-of-Cure

In the micro-ITT population, exploratory analysis of microbiological response determined by NAAT at the test-of-cure showed that the treatment success rate for urogenital *N. gonorrhoeae* numerically favored gepotidacin compared with ceftriaxone plus azithromycin (82.5% [151/183] vs 75.0% [132/176]; adjusted difference 8.2%, 95% CI −0.1%, 16.5%; Table 3). Failure due to nucleic acid persistence of urogenital *N. gonorrhoeae* was observed at similar rates with gepotidacin (10.9% [20/183]) and ceftriaxone plus azithromycin (11.9% [21/176]), while failure rates due to “unable to determine” outcome were numerically lower with gepotidacin compared with ceftriaxone plus azithromycin (6.6% [12/183] vs 13.1% [23/176]; Table 3). Reasons for the “unable to determine” outcome included improper specimen submission, specimens not being collected, and participants not returning for the test-of-cure visit. No bacterial persistence was observed at the urogenital site with either treatment, as determined by microbiologic culture.

**Table 3.** Summary of Microbiological Outcome and Treatment Response for Urogenital *Neisseria gonorrhoeae* at Test-of-Cure Determined by NAAT (Micro-ITT and Microbiologically Evaluable NAAT Test-of-Cure Populations)

	Micro-ITT^a^		Microbiologically Evaluable NAAT Test-of-Cure	
Treatment Response (Determined by NAAT) Microbiological Outcome	Gepotidacin 2 × 3000 mg (N = 183)	Ceftriaxone 500 mg Plus Azithromycin 1 g (N = 176)	Gepotidacin 2 × 3000 mg (N = 171)	Ceftriaxone 500 mg Plus Azithromycin 1 g (N = 153)
Treatment success, n (%)	151 (82.5)	132 (75.0)	151 (88.3)	132 (86.3)
95% CI for treatment success^b^	(76.2%, 87.7%)	(67.9%, 81.2%)	(82.5%, 92.7%)	(79.8%, 91.3%)
Success rate difference^c^ (95% CI)	8.2% (−0.1%, 16.5%)		3.8% (−3.6%, 11.2%)	
Treatment failure, n (%)	32 (17.5)	44 (25.0)	20 (11.7)	21 (13.7)
Nucleic acid persistence	20 (10.9)	21 (11.9)	20 (11.7)	21 (13.7)
Unable to determine	12 (6.6)	23 (13.1)	0	0

Abbreviations: CI, confidence interval; micro-ITT, microbiological intent-to-treat; NAAT, nucleic acid amplification test.

^a^A total of 406 participants in the micro-ITT population provided specimens, 202 in the gepotidacin arm and 204 in the ceftriaxone plus azithromycin arms, respectively; N is the number of participants who were *N. gonorrhoeae* NAAT positive at baseline at the urogenital body site.

^b^Exact Clopper–Pearson CI.

^c^Success rate difference (percentage of success in the gepotidacin group minus the percentage of success in the ceftriaxone/azithromycin group) using Miettinen–Nurminen (score) method, adjusting for treatment and pooled actual strata of sex and sexual orientation (men who have sex with women; men who have sex with men, including bisexual male participants who reported sex with both men and women; or female) for the urogenital body site.

Exploratory analysis of microbiological response by NAAT in the microbiologically evaluable NAAT test-of-cure population showed a similar treatment success rate for urogenital *N. gonorrhoeae* in both treatment groups (88.3% [151/171] gepotidacin, 86.3% [132/153] ceftriaxone plus azithromycin; adjusted difference 3.8%, 95% CI, −3.6%, 11.2%; Table 3). Failure rates due to nucleic acid persistence for urogenital *N. gonorrhoeae* were similar with gepotidacin (11.7% [20/171]) and ceftriaxone plus azithromycin (13.7% [21/153]). Nucleic acid amplification test positive results for *N. gonorrhoeae* were observed on each day of the test-of-cure analysis visit window (days 4–10) for both treatment arms, with the exception of day 9 in the gepotidacin group (post hoc analysis, Table 4).

**Table 4.** Summary of Culture and NAAT Identification of Urogenital *Neisseria gonorrhoeae* at the Test-of-Cure Visit by Analysis Day in the Microbiologically Evaluable NAAT Test-of-Cure Population (Post Hoc Analysis)

	Test-of-Cure Study Day,^a^ n^b^ (%)							
	Day 4	Day 5	Day 6	Day 7	Day 8	Day 9	Day 10	Total^c^
Gepotidacin 2 × 3000 mg								
Culture	0/10	0/22	0/16	0/43	0/68	0/8	0/4	0/171
NAAT	1/10 (10.0)	3/22 (13.6)	2/16 (12.5)	7/43 (16.3)	6/68 (8.8)	0/8	1/4 (25.0)	20/171 (11.7)
Ceftriaxone 500 mg plus azithromycin 1 g								
Culture	0/6	0/17	0/30	0/31	0/53	0/12	0/4	0/153
NAAT	2/6 (33.3)	3/17 (17.6)	5/30 (16.7)	2/31 (6.5)	7/53 (13.2)	1/12 (8.3)	1/4 (25.0)	21/153 (13.7)
Total								
Culture	0/16	0/39	0/46	0/74	0/121	0/20	0/8	0/324
NAAT	3/16 (18.8)	6/39 (15.4)	7/46 (15.2)	9/74 (12.2)	13/121 (10.7)	1/20 (5.0)	2/8 (25.0)	41/324 (12.7)

Abbreviation: NAAT, nucleic acid amplification test.

^a^The test-of-cure visit window was days 4–8 per the study protocol, and the analysis visit window was days 4–10 per the study reporting and analysis plan.

^b^n is calculated as the total number of positive samples divided by the total number of samples.

^c^Total is the sum of results during the analysis window (days 4–10). Results outside this window are not included.

### Rectal and Pharyngeal Sites: Treatment Efficacy by NAAT

In the micro-ITT rectal population at the test-of-cure, the treatment success rate for rectal *N. gonorrhoeae*, determined by NAAT, numerically favored gepotidacin (96.0% [24/25] vs 64.3% [9/14]; difference 31.7%, 95% CI, 8.1%, 58.4%; Supplementary Table 4). There were no failures due to nucleic acid persistence or by culture with either treatment.

The treatment success rate at the test-of-cure visit (determined by NAAT) for pharyngeal *N. gonorrhoeae* in the micro-ITT pharyngeal population numerically favored ceftriaxone plus azithromycin (53.3% [8/15] vs 81.3% [13/16]; difference −27.9%, 95% CI, −56.5%, 5.6%; Supplementary Table 5). In the microbiologically evaluable NAAT test-of-cure pharyngeal population, treatment failure rates due to nucleic acid persistence determined by NAAT were 40.0% (6/15) and 12.5% (2/16) for the gepotidacin and ceftriaxone plus azithromycin treatment arms, respectively. Bacterial persistence determined by culture was observed in 2 participants in the gepotidacin group and none in the ceftriaxone plus azithromycin group; both gepotidacin participants with bacterial persistence by culture were also NAAT positive at the test-of-cure.

Very few pharyngeal *N. gonorrhoeae* specimens were available for evaluating microbiological response by NAAT at the follow-up visit, limiting interpretation (4 gepotidacin and 2 ceftriaxone plus azithromycin; micro-ITT pharyngeal population). Two participants in the gepotidacin treatment group, but no participants in the ceftriaxone plus azithromycin group, had pharyngeal nucleic acid persistence at the follow-up visit. One culture-positive specimen was identified at the pharyngeal site in 1 participant at follow-up; this participant was also NAAT positive. Full outcomes in the micro-ITT rectal, micro-ITT pharyngeal, and microbiological evaluable populations for each body site are presented in Supplementary Results and Supplementary Tables 4–6.

## DISCUSSION

Primary efficacy results of the phase 3 EAGLE-1 study were based on microbiologic culture and demonstrated that oral gepotidacin was noninferior to ceftriaxone plus azithromycin for the treatment of uncomplicated urogenital gonorrhea [15]. Herein, exploratory analyses using NAAT for evaluation of efficacy demonstrated that microbiological success at the urogenital site at the test-of-cure (days 4–8) was high and similar between gepotidacin (82.5%) and ceftriaxone plus azithromycin (75.0%; micro-ITT population). This supports the primary analysis based on microbiological culture, in which microbiological success rates were high and there was no bacterial persistence observed at the urogenital site in either treatment arm.

Test-of-cure in EAGLE-1 was scheduled on days 4–8, in agreement with FDA guidelines, to optimize the detection of *N. gonorrhoeae* by culture. As expected, overall urogenital success rates with NAAT were numerically lower than the culture-defined success rates, given evidence that gonococcal DNA may continue to be present in the absence of viable bacteria (ie, culture negative) for 14 days or more following treatment [24–27]; UK guidelines, therefore, recommend that a test-of-cure using NAATs should be performed ≥14 days after treatment [5]. A negative NAAT at the test-of-cure may, however, be informative, notably in the event of a missing or unable to determine culture result.

Microbiologic culture and/or NAATs are recommended for the diagnosis of gonorrhea [4–7]. In our exploratory analyses of EAGLE-1, we found high concordance between NAAT and culture for urogenital *N. gonorrhoeae* identification at baseline. While no currently available test for *N. gonorrhoeae* offers 100% sensitivity and specificity [5], these data support NAATs as a complementary tool to culture in future registrational trials.

Collection of rectal and pharyngeal specimens was optional and required separate patient consent, resulting in considerably smaller sample sizes at these body sites. By NAAT, treatment success rates at the rectal site numerically favored gepotidacin over ceftriaxone plus azithromycin. At the pharyngeal site, the NAAT-determined treatment failure rate was numerically higher for gepotidacin; 1 participant was both culture and NAAT positive at follow-up, compared with the ceftriaxone plus azithromycin arm in which no participants were culture or NAAT positive at follow-up. The anatomical structure of the pharynx and characteristics of pharyngeal tissue often result in suboptimal antibiotic concentrations, making pharyngeal *N. gonorrhoeae* infections more prone to treatment failure in general [28–30].

This work has some limitations. Firstly, the timing of test-of-cure evaluation was optimized for culture-based assessment, rather than for evaluation by NAAT assay, due to US FDA regulatory requirements (the primary efficacy endpoint should be established 3–7 days after receipt of treatment [17]) that mandated culture assessment at days 4–8 be used in the EAGLE-1 study [15]. Efficacy data for *N. gonorrhoeae* infection at the rectal and pharyngeal sites were based on a small number of specimens, collection of which required separate patient consent and was optional to reduce burden on participants and mitigate discomfort. These results should thus be interpreted with caution, and further research is needed to evaluate efficacy based on NAAT results at nonurogenital sites. Additionally, consistent with FDA guidance for noninferiority trials, only participants with ceftriaxone-susceptible isolates were included to avoid bias toward the investigational drug and poor comparator performance; therefore, evaluation of gepotidacin efficacy among patients with gonorrhea caused by ceftriaxone-resistant strains is warranted. Notably, all isolates recovered at baseline were susceptible to ceftriaxone, regardless of body site, and no patients were excluded based on ceftriaxone nonsusceptibility. A further limitation of the work presented is the lack of central laboratory culture-negative data at baseline, which prohibited assessment of the specificity of NAAT at baseline, and similarly the high microbiological success rates (ie, very little culture-positive data) at the test-of-cure prohibited assessment of sensitivity and false positives with NAATs at the test-of-cure.

In conclusion, treatment success rates determined by NAAT at the urogenital site were high and similar following treatment with gepotidacin or ceftriaxone plus azithromycin, supporting the primary analysis based on culture. This was despite the timing of test-of-cure assessment in EAGLE-1 having been optimized for microbiologic culture rather than NAAT (per regulatory requirements). With similar efficacy conclusions between NAAT and culture, NAAT may be a promising addition to culture in future registrational urogenital gonorrhea clinical trials, offering rapid testing with greater practicality and diagnostic sensitivity to complement the antimicrobial susceptibility profiling provided by culture.

## Supplementary Material

ofag438_Supplementary_Data

## Supplementary Data

Supplementary materials are available at *Open Forum Infectious Diseases* online. Consisting of data provided by the authors to benefit the reader, the posted materials are not copyedited and are the sole responsibility of the authors, so questions or comments should be addressed to the corresponding author.
