# Supplementary material for: Oral Gepotidacin for the Treatment of Uncomplicated Urogenital Gonorrhea: Nucleic Acid Amplification Testing Outcomes in a Randomized, Multicenter Phase 3 Trial (EAGLE-1)
Source: Open Forum Infect Dis. 2026 Jul 17;13(7):ofag438. doi: 10.1093/ofid/ofag438 (PMC13403078; doi:10.1093/ofid/ofag438)
Supplement: ofag438_Supplementary_Data [file ofag438_supplementary_data.docx]

**Supplementary Material**

**Title:** Oral gepotidacin for the treatment of uncomplicated urogenital gonorrhea: nucleic acid amplification testing (NAAT) outcomes in a randomized, multicenter phase 3 trial (EAGLE-1)

**Authors**

| **Name** | **Affiliation** | **Highest degree** |
| --- | --- | --- |
| William G. Flight | GSK, London, UK | MD, PhD |
| Janet Wilson | The Leeds Teaching Hospitals NHS Trust, Leeds, UK | MB ChB |
| David A. Lewis | Sydney Medical School-Westmead, University of Sydney, Sydney, NSW, Australia  Western Sydney Sexual Health Centre, Western Sydney Local Health District, Sydney, NSW, Australia | PhD |
| Jonathan Ross | University Hospitals Birmingham NHS Foundation Trust, Birmingham, UK | MD |
| Sally Gatsi | Organon LLC, Jersey City, NJ, USA | PharmD |
| Charles Jakielaszek | GSK, Collegeville, PA, USA | BSc |
| Dan T. Lythgoe | GSK, Stevenage, UK | PhD |
| Salim Janmohamed | GSK, London, UK | MBBS (Hons) |
| Judith Absalon | GSK, Collegeville, PA, USA | MD |
| Matthew Helgeson | GSK, Collegeville, PA, USA | PharmD |
| Caroline Perry | GSK, Collegeville, PA, USA | PhD |

**Corresponding author:** William Flight, GSK, London, UK; email: [william.g.flight@gsk.com](mailto:william.g.flight@gsk.com)

**Supplementary Methods**

**Efficacy – microbiological success**

Clearance of baseline *N. gonorrhoeae* nucleic acid was also assessed in: pharyngeal specimens at the test-of-cure and follow-up visits; and rectal specimens at the test-of-cure visit.

**Analysis populations**

Two subsets of the microbiological intent-to-treat (micro-ITT) population were also examined based on confirmed culture of ​ceftriaxone-susceptible *N. gonorrhoeae* at baseline at the rectal (micro-ITT rectal population) and pharyngeal (micro-ITT pharyngeal population) body sites.

Two subsets of the microbiologically evaluable nucleic acid amplification test (NAAT) test-of-cure population were evaluated: the microbiologically evaluable NAAT test-of-cure rectal population and the microbiologically evaluable NAAT test-of-cure pharyngeal population. These comprised participants who met the definitions of the micro-ITT rectal or pharyngeal populations, respectively, were NAAT-positive at the corresponding site at baseline, and who also met the same important components of the study as the main microbiologically evaluable NAAT test-of-cure population.

**Statistical analysis**

For efficacy analyses based on the rectal and pharyngeal body sites, the unadjusted difference in microbiological success rates was calculated and summarized along with the corresponding two-sided 95% confidence intervals (CI) using the Miettinen–Nurminen score method (no adjustment for strata) [1].

**Supplementary Results**

**Study disposition**

The micro-ITT rectal population included 41 participants (26 gepotidacin; 15 ceftriaxone plus azithromycin), of which 33 (24 gepotidacin; nine ceftriaxone plus azithromycin) comprised the microbiologically evaluable NAAT test-of-cure rectal population. The micro-ITT pharyngeal population included 35 participants (18 gepotidacin; 17 ceftriaxone plus azithromycin), of which 29 participants (14 gepotidacin; 15 ceftriaxone plus azithromycin) comprised the microbiologically evaluable NAAT test-of-cure pharyngeal population.

**Rectal site – treatment efficacy by NAAT at test-of-cure**

In the microbiologically evaluable NAAT test-of-cure rectal population, treatment success rates determined by NAAT for rectal *N. gonorrhoeae* at test-of-cure were 100% in both treatment groups (24/24 with gepotidacin and 9/9 with ceftriaxone plus azithromycin; **Supplementary Table 5**). For context, there were no culture positives observed at test-of-cure at the rectal site with either treatment.

**Pharyngeal site – treatment efficacy by NAAT at test-of-cure**

The success rate for pharyngeal *N. gonorrhoeae* numerically favored ceftriaxone plus azithromycin in the microbiologically evaluable NAAT test-of-cure pharyngeal population (57.1% [8/14] vs 86.7% [13/15]; difference –29.5%, 95% CI, –58.2%, 3.8%) (**Supplementary Table 6***).*

In a post-hoc analysis of the microbiologically evaluable NAAT test-of-cure pharyngeal population, NAAT was positive in one participant on day 4, four participants on day 7 and one participant on day 8 with gepotidacin; NAAT was positive in one participant on day 6 and one participant on day 7 with ceftriaxone plus azithromycin (**Supplementary Table 7**). For context, two culture-positives were identified at the pharyngeal site in two participants treated with gepotidacin and none for participants treated with ceftriaxone plus azithromycin. Sample sizes for the pharyngeal populations were small, limiting any interpretation of differences between treatment groups.

**Supplementary Tables**

**Supplementary Table 1.** Summary of culture collection by body site and visit

| **Body site** | **Culture type** | **Baseline visit** | **Test-of-cure visit** | **Follow-up visit** |
| --- | --- | --- | --- | --- |
| Urogenital | *N. gonorrhoeae* | X | X |  |
| Pharyngeal^a^ | *N. gonorrhoeae* | X | X | X^b^ |
| Rectal^a^ | *N. gonorrhoeae* | X | X |  |

^a^Optional (upon patient consent).

^b^Only if positive NAAT at baseline and test-of-cure visit.

Abbreviations: NAAT, nucleic acid amplification test; *N. gonorrhoeae*, *Neisseria gonorrhoeae*.

**Supplementary Table 2.**  Summary of *N. gonorrhoeae* NAAT assessments by body site and visit

| **Body site** | **Baseline visit** | **Test-of-cure visit** | **Follow-up visit** |
| --- | --- | --- | --- |
|  | **Central laboratory** | **Central laboratory** | **Central laboratory** |
| Urogenital | X | X |  |
| Pharyngeal^a^ | X | X | X^b^ |
| Rectal^a^ | X | X |  |

^a^Optional (upon patient consent).

^b^Only if NG positive NAAT at baseline and test-of-cure visit.

Abbreviations: NAAT, nucleic acid amplification test; *N*. *gonorrhoeae*, *Neisseria gonorrhoeae*.

**Supplementary Table 3.** Concordance between central laboratory culture identification and central laboratory NAAT at baseline visit^a^ at the urogenital site for both treatment arms (post-hoc analysis)

|  | **Culture positive** | **Culture negative** | **Total (N=371)** |
| --- | --- | --- | --- |
| **NAAT positive** | 360 (97.0%) | 4 (1.1%) | 364 |
| **NAAT negative** | 2 (0.5%) | 5 (1.3%) | 7 |

^a^Excluding UTD and missing data. n is the number of observations for which both baseline central laboratory NAAT and central laboratory culture results were available (ie, Unable to Determine/Missing/Not Done results excluded). Overall concordance, the percentage of observations which were concordant between the methods, was 98.4%. Sensitivity, the percentage of culture-positive observations which were also NAAT-positive, was 99.4%. Positive predictive value, the percentage of NAAT-positive observations which were also culture-positive was 98.9%.

Abbreviations: NAAT, nucleic acid amplification test; *N. gonorrhoeae*, *Neisseria gonorrhoeae*; UTD, unable to determine.

**Supplementary Table 4****.** Summary of microbiological outcome and treatment response for rectal *N. gonorrhoeae* at test-of-cure, determined by NAAT (micro-ITT rectal and microbiologically evaluable NAAT test-of-cure rectal populations)

|  | **Micro-ITT rectal^a^** | | **Microbiologically evaluable NAAT test-of-cure rectal** | |
| --- | --- | --- | --- | --- |
| **Treatment response (determined by NAAT)**  **Microbiological outcome** | **Gepotidacin**  **2x3000 mg^b^**  **(N=25)** | **Ceftriaxone 500 mg**  **plus azithromycin 1 g**  **(N=14)** | **Gepotidacin**  **2x3000 mg**^b^  **(N=24)** | **Ceftriaxone 500 mg**  **plus azithromycin 1 g**  **(N=9)** |
| Treatment Success | 24 (96.0%) | 9 (64.3%) | 24 (100%) | 9 (100%) |
| 95% CI for Treatment Success^c^ | (79.6%, 99.9%) | (35.1%, 87.2%) | (85.8%, 100%) | (66.4%, 100%) |
| Success Rate Difference^d^ | 31.7% (8.1%, 58.4%) | | 0 (–14.2%, 30.6%) | |
| Treatment Failure | 1 (4.0%) | 5 (35.7%) | 0 | 0 |
| Nucleic acid Persistence | 0 | 0 | 0 | 0 |
| Unable to Determine | 1 (4.0%) | 5 (35.7%) | 0 | 0 |

^a^A total of 41 participants in the micro-ITT rectal population provided specimens (26 and 15 participants in the gepotidacin and ceftriaxone plus azithromycin arms, respectively); N is the number of participants who were *N. gonorrhoeae* NAAT positive at baseline at the rectal body site.

^b^The gepotidacin dose selection for this study was optimized for the urogenital body site, not for the rectal body site.

^c^Exact Clopper-Pearson CI.

^d^Success Rate Difference (gepotidacin minus ceftriaxone/azithromycin) using unadjusted Miettinen-Nurminen method for the rectal body site.

Abbreviations: CI, confidence interval; Micro-ITT, microbiological intent-to-treat; *N. gonorrhoeae*, *Neisseria gonorrhoeae*; NAAT, nucleic acid amplification test.

**Supplementary Table 5.** Summary of microbiological outcome and treatment response for pharyngeal *N. gonorrhoeae* at test-of-cure determined by NAAT (micro-ITT pharyngeal and microbiologically evaluable NAAT test-of-cure pharyngeal populations)

|  | **Micro-ITT pharyngeal^a^** | | | **Microbiologically evaluable NAAT test-of-cure pharyngeal** | |
| --- | --- | --- | --- | --- | --- |
| **Treatment response  (determined by NAAT)**  **Microbiological outcome** | **Gepotidacin**  **2 x 3000 mg^b^**  **(N=15)** | **Ceftriaxone 500 mg**  **plus azithromycin 1 g**  **(N=16)** | | **Gepotidacin**  **2 x 3000 mg^b^**  **(N=14)** | **Ceftriaxone 500 mg**  **plus azithromycin 1 g**  **(N=15)** |
| Treatment Success | 8 (53.3%) | 13 (81.3%) | | 8 (57.1%) | 13 (86.7%) |
| 95% CI for Treatment Success^c^ | (26.6%, 78.7%) | (54.4%, 96.0%) | | (28.9%, 82.3%) | (59.5%, 98.3%) |
| Success Rate Difference^d^ | –27.9% (–56.5%, 5.6%) | | | –29.5% (–58.2%, 3.8%) | |
| Treatment Failure | 7 (46.7%) | | 3 (18.8%) | 6 (42.9%) | 2 (13.3%) |
| Nucleic acid Persistence | 6 (40.0%) | | 2 (12.5%) | 6 (42.9%) | 2 (13.3%) |
| Unable to Determine | 1 (6.7%) | | 1 (6.3%) | 0 | 0 |

^a^A total of 35 participants in the micro-ITT pharyngeal population provided specimens (18 and 17 in the gepotidacin and ceftriaxone plus azithromycin arms, respectively); N is the number of participants who were *N. gonorrhoeae* NAAT positive at baseline at the pharyngeal body site.

^b^The gepotidacin dose selection for this study was optimized for the urogenital body site, not for the pharyngeal body site.

^c^Exact Clopper-Pearson CI.

^d^Success Rate Difference (gepotidacin minus ceftriaxone/azithromycin) using unadjusted Miettinen-Nurminen method for the pharyngeal body site.

Abbreviations: CI, confidence interval; Micro-ITT, microbiological intent-to-treat; *N. gonorrhoeae*, *Neisseria gonorrhoeae*; NAAT, nucleic acid amplification test.

**Supplementary Table 6.** Summary of culture and NAAT identification of *N. gonorrhoeae* at the test-of-cure visit by analysis day in the microbiologically evaluable NAAT test-of-cure pharyngeal population (post-hoc analysis)

|  | **Test-of-cure study day,^a^ n^b^ (%)** | | | | | | | |
| --- | --- | --- | --- | --- | --- | --- | --- | --- |
|  | **Day 4** | **Day 5** | **Day 6** | **Day 7** | **Day 8** | **Day 9** | **Day 10** | **Total^c^** |
| **Gepotidacin 2 × 3000 mg** | | | | | | | | |
| Culture | 0/1 | 0/0 | 0/1 | 2/5 (40.0%) | 0/5 | 0/1 | 0/1 | 2/14 (14.3%) |
| NAAT | 1/1 (100.0%) | 0/0 | 0/1 | 4/5 (80.0%) | 1/5 (20.0%) | 0/1 | 0/1 | 6/14 (42.9%) |
| **Ceftriaxone 500 mg plus azithromycin 1 g** | | | | | | | | |
| Culture | 0/0 | 0/1 | 0/4 | 0/4 | 0/5 | 0/1 | 0/0 | 0/15 |
| NAAT | 0/0 | 0/1 | 1/4 (25.0%) | 1/4 (25.0%) | 0/5 | 0/1 | 0/0 | 2/15 (13.3%) |
| **Total** | | | | | | | | |
| Culture | 0/1 | 0/1 | 0/5 | 2/9 (22.2%) | 0/10 | 0/2 | 0/1 | 2/29 (6.9%) |
| NAAT | 1/1 (100.0%) | 0/1 | 1/5 (20.0%) | 5/9 (55.6%) | 1/10 (10.0%) | 0/2 | 0/1 | 8/29 (27.6%) |

^a^The test-of-cure visit window was day 4–8 per the study protocol and the analysis visit window was day 4–10 per the study reporting and analysis plan.

^b^n is calculated as the total number of positive samples divided by the total number of samples.

^c^Total is the sum of results during the analysis window (day 4–10). Results outside this window are not included.

Abbreviations: NAAT, nucleic acid amplification test; *N. gonorrhoeae*, *Neisseria gonorrhoeae.*

**References**

1. Miettinen O, Nurminen M. Comparative analysis of two rates. Stat Med **1985**; 4(2): 213–26.
